# Evaluation of a High-Throughput Peptide Reactivity Format Assay for Assessment of the Skin Sensitization Potential of Chemicals

**DOI:** 10.3389/fphar.2016.00053

**Published:** 2016-03-14

**Authors:** Chin Lin Wong, Ai-Leen Lam, Maree T. Smith, Sussan Ghassabian

**Affiliations:** ^1^Centre for Integrated Preclinical Drug Development, The University of QueenslandSt Lucia, QLD, Australia; ^2^School of Pharmacy, The University of QueenslandWoolloongabba, QLD, Australia

**Keywords:** allergic contact dermatitis, Cor1-C420, cysteine, *in vitro* methods, lysine, peptide reactivity assay, skin sensitization

## Abstract

The direct peptide reactivity assay (DPRA) is a validated method for *in vitro* assessment of the skin sensitization potential of chemicals. In the present work, we describe a peptide reactivity assay using 96-well plate format and systematically identified the optimal assay conditions for accurate and reproducible classification of chemicals with known sensitizing capacity. The aim of the research is to ensure that the analytical component of the peptide reactivity assay is robust, accurate, and reproducible in accordance with criteria that are used for the validation of bioanalytical methods. Analytical performance was evaluated using quality control samples (QCs; heptapeptides at low, medium, and high concentrations) and incubation of control chemicals (chemicals with known sensitization capacity, weak, moderate, strong, extreme, and non-sensitizers) with each of three synthetic heptapeptides, viz Cor1-C420 (Ac-NKKCDLF), cysteine- (Ac-RFAACAA), and lysine- (Ac-RFAAKAA) containing heptapeptides. The optimal incubation temperature for all three heptapeptides was 25°C. Apparent heptapeptide depletion was affected by vial material composition. Incubation of test chemicals with Cor1-C420, showed that peptide depletion was unchanged in polypropylene vials over 3-days storage in an autosampler but this was not the case for borosilicate glass vials. For cysteine-containing heptapeptide, the concentration was not stable by day 3 post-incubation in borosilicate glass vials. Although the lysine-containing heptapeptide concentration was unchanged in both polypropylene and borosilicate glass vials, the apparent extent of lysine-containing heptapeptide depletion by ethyl acrylate, differed between polypropylene (24.7%) and glass (47.3%) vials. Additionally, the peptide-chemical complexes for Cor1-C420-cinnamaldehyde and cysteine-containing heptapeptide-2, 4-dinitrochlorobenzene were partially reversible during 3-days of autosampler storage. These observations further highlight the difficulty in adapting *in vitro* methods to high-throughput format for screening the skin sensitization potential of large numbers of chemicals whilst ensuring that the data produced are both accurate and reproducible.

## Introduction

Allergic contact dermatitis (ACD) is the clinically significant consequence of skin sensitization that negatively affects ~15–20% of the general population (Peiser et al., 2012). At present, more than 4000 chemicals are linked to induction of ACD in humans (Cahill et al., 2012). A number of contact allergens, including fragrances, epoxy resin systems, formaldehyde, neomycin sulfate, and nickel sulfate are commonly reported to induce ACD in humans (Cahill et al., 2012; Pesonen et al., 2015).

Due to the high incident rate of ACD in general population, several animal models such as guinea pig test (Buehler, 1965; Magnusson and Kligman, 1969, 1970; Magnusson et al., 1979; Magnusson, 1980) and murine local lymph node assay (LLNA; Kimber and Basketter, 1992; Kimber et al., 1994, 1998; Basketter et al., 1996) have been developed. However, according to the European Cosmetic Directive (EC1223/2009), the ban of cosmetic ingredients that were subjected to animal testing was enforced. Moreover, finished cosmetic products subjected to animal testing were prohibited from being marketed in the European Union (EU) since 2009 (EU, 2009). Nevertheless, animal testing was still allowed for determining the complex human health effect. On 11 March 2013, full ban on animal testing for cosmetic purposes was enforced. Besides, the EU REACH regulation (registration, evaluation, authorization, and restriction of chemicals), EC1907/2006, that came into force on 1 June 2007, provided a strong imperative for the development and implementation of rapid *in vitro* screening methods (EU, 2006) for assessing the skin sensitization potential of chemicals that had not been previously tested using *in vivo* methods. Furthermore, implementation of the 3Rs, reduction, refinement and replacement of animal testing, has driven the need to adopt alternative non-animal skin sensitization screening methods. Validated *in vitro* methods are thus essential for identifying potential skin sensitizers to prevent ACD (EU, 2009).

To this end, multiple non-animal testing methods have been developed and evaluated. For examples, direct peptide reactivity assay (DPRA), human cell line activation test (h-CLAT), KeratinoSens™, and myeloid U937 skin sensitization test (MUSST; Gerberick et al., 2004; Ade et al., 2006; Ashikaga et al., 2006; Sakaguchi et al., 2006; Python et al., 2007; Emter et al., 2010; Bauch et al., 2012). It was anticipated that *in vitro* methods would have the ability to assess hundreds of chemicals concurrently, which is not feasible with methods utilizing the murine LLNA. Although the mouse LLNA was generally regarded as the “gold standard” test system for identifying skin sensitization potential of contact allergens (Kimber et al., 1986; Kimber and Weisenberger, 1989; ICCVAM, 1998; Dean et al., 2001; OECD, 2010), the findings did not necessarily always correlate well with human data due to variability of the test (Kolle et al., 2013; Basketter et al., 2014; Hoffmann, 2015) and it has been shown that the *in vitro* tests predict human sensitizing potential better than the LLNA (Urbisch et al., 2015). This may be due to inter-species differences in anatomy, physiology, biochemistry, and immunology that underpin differential skin responses to various chemicals between mice and humans (Jamei et al., 2009).

The DPRA is adopted by the Organization for Economic Co-operation and Development (OECD) for use in the hazard assessment of chemicals as potential skin sensitizers (OECD, 2012). The ability of haptens to bind with skin proteins is regarded as the initial key event in skin sensitization (OECD, 2013). Hapten-protein complexes are formed via covalent modification of amino acid side chains of proteins. This process, known as haptenation, provides the scientific basis underpinning the DPRA (Gerberick et al., 2004, 2007). Most sensitizing chemicals are electrophilic in nature, comprising Michael acceptors, S_N_Ar and S_N_2 electrophiles, Schiff base formers, or acylating agents and so possess the ability to react with the nucleophilic amino acid residues of skin proteins (Chipinda et al., 2011; Lalko et al., 2012). While lysine- and cysteine-containing heptapeptides more commonly bind covalently to these electrophiles, other residues such as histidine and methionine also react with haptens (Gerberick et al., 2009). Irreversible covalent bond formation between haptens and amino acid residues of skin proteins is mimicked in the DPRA whereby the amount of unreacted exogenous peptide is quantified in the presence and absence of potential skin sensitizing chemicals (Gerberick et al., 2004).

The sensitivity and accuracy of various amino acid combinations for simulation of skin proteins in the peptide reactivity assay have been investigated. Gerberick et al. (2007) proposed the optimum combinations of the amino acids, glutathione-, cysteine,- and lysine-containing peptides for accurately identifying skin sensitizers. The overarching principle was to eliminate the need for utilization of a large panel of peptides to ensure reliability of the DPRA for predictive purposes (Gerberick et al., 2007). Based upon this approach, peptides containing cysteine or lysine, at a 1:10 or 1:50 molar ratio to the test chemicals of interest, respectively, were found to give the best predictive power for the DPRA (Gerberick et al., 2007). In addition, a synthetic peptide containing both cysteine and lysine residues (Cor1-C420) which had the added advantage of high aqueous solubility in reaction buffer, showed high reactivity toward electrophiles (Dennehy et al., 2006; Natsch et al., 2007) and had previously shown promising results for identification of skin sensitizers (Natsch and Gfeller, 2008). Recently, several improvements for peptide reactivity assay has been proposed as reviewed in Wong et al. (2015). Due to the nature of chemical reactivity, it is crucial to incorporate several peptides in the peptide reactivity assay. Nevertheless, it is important to identify the optimum conditions for each peptide under different circumstances.

To minimize inter and intra-laboratory variability in peptide reactivity results, it is important to ensure that the analytical component of the method is robust, accurate, and reproducible in accordance with criteria that are used for the validation of bioanalytical methods (EMA, 2011; FDA, 2011). Hence, our aims were to develop, optimize, and assess the performance of three liquid chromatography tandem mass spectrometry (LC-MS/MS) analytical methods for quantification of the concentrations of lysine- and cysteine-containing heptapeptides as well as Cor1-C420 heptapeptides, following their reaction with various representative test chemicals of known skin sensitization potential. Analytical method validation parameters include accuracy, precision, carry-over, stability of peptides under various incubation temperatures, influence of solvent composition, autosampler stability over 72 h, and impact of vial materials on assay performance. No direct comparison with the OECD guideline test or testing of the substances in its minimum performance standard was carried out in this paper.

## Materials and methods

### Peptides

Leucine enkephalin acetate salt hydrate (YGGFL) (>98%) was supplied by Sigma-Aldrich Corporation (NSW, Australia), α-N-acetyl leucine enkephalin (Ac-YGGFL) (>95%), cysteine-containing heptapeptides (Ac-RFAACAA) (>94%), lysine-containing heptapeptides (Ac-RFAAKAA) (>97%), and Cor1-C420 (Ac-NKKCDLF) (>98%) heptapeptides were supplied by GL Biochem (Shanghai, China).

### Chemicals and reagents

1-chloro-2,4-dinitrobenzene (DNCB, 99.8%, CAS 97-00-7), cinnamaldehyde (98.4%, CAS 104-55-2), ethyl acrylate (100%, CAS 140-88-5), glutaraldehyde (25%, CAS 111-30-8), isoeugenol (99%, CAS 97-54-1), methyl salicylate (99.4%, CAS 119-36-8), ammonium hydroxide solution (28–30%), bovine serum albumin (BSA), DL-dithiothreitol (DTT), and deferoxamine mesylate salt were supplied by Sigma-Aldrich Corporation (NSW, Australia), high performance liquid chromatography (HPLC) grade methanol and acetonitrile were supplied by Merck (Darmstadt, Germany), sodium hydroxide and ammonium acetate were supplied by Chem-Supply (SA, Australia). Sodium phosphate dibasic and monosodium phosphate were purchased from ThermoFisher Scientific (VIC, Australia).

### Experimental design

#### LC conditions

**Figure 1:**
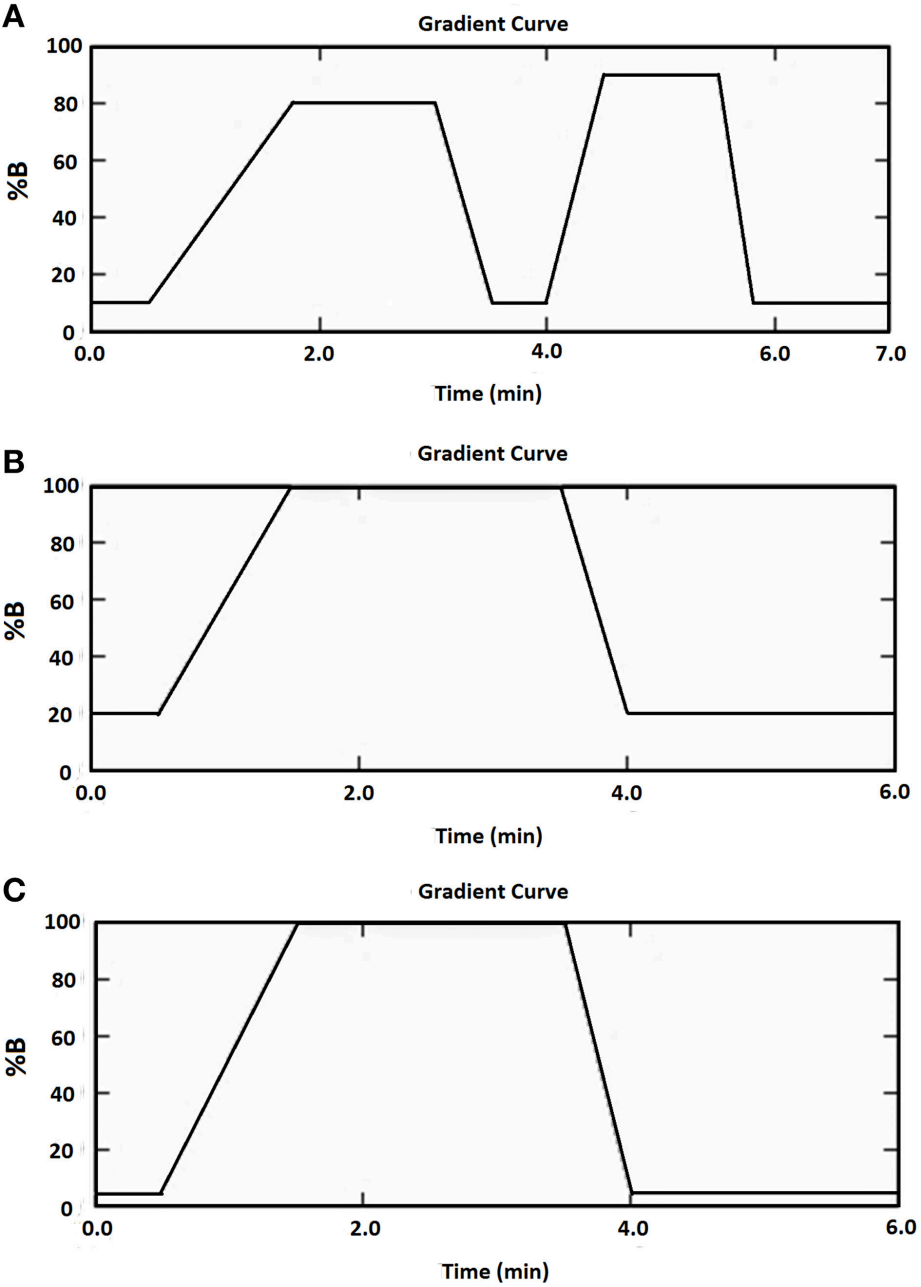
**Mobile phase gradient elution of (A) Cor1-C420 (B) cysteine- (C) lysine-containing heptapeptides**.

The HPLC apparatus was a Shimadzu chromatographic system. A reversed phase C18 column (Gemini, 2.0 × 150 mm, particle size 5 μm; Phenomenex, NSW, Australia) and a C18 security guard column (Gemini, Phenomenex, NSW, Australia) was used for all three heptapeptides. The column oven and autosampler temperatures were set at 40 and 4°C, respectively. The injection volume for all samples was 5 μL. The mobile phase for the Cor1-C420 heptapeptides comprised mobile phase A (10 mM ammonium acetate, pH 9.5) and mobile phase B (acetonitrile) and the flow rate was 0.4 m/min. The mobile phases for the heptapeptides containing cysteine or lysine comprised mobile phase A (10 mM ammonium acetate, pH 9.5) and mobile phase B (methanol) and the flow rate was 0.5 mL/min. A stepwise gradient elution program summarized in Figure 1 was used for each heptapeptide. The acquisition and processing of data were performed using the Applied Biosystems Sciex Analyst™ software, version 1.6.1.

#### MS/MS conditions

MS detection was carried out using an Applied Biosystems Sciex API 3200 triple quadruple MS equipped with an electrospray ionization source. The highest abundance product ions were selected for each analyte. Positive ionization mode was chosen for all three heptapeptides and the corresponding internal standards. The first 5 min of the chromatographic run time were acquired by the MS. To tune the parameters for the heptapeptides and internal standards, molecular ions were identified by direct infusion of the solutions of interest and the parameters were automatically acquired by the Analyst™ software version 1.6.1. Multiple reaction monitoring (MRM) in positive ionization mode was used to monitor the analytes. The MS parameters for each heptapeptide and internal standard are listed in Table 1. The chromatographic methods and peak area integrations were performed using Analyst™ software.

**Table 1:** **MS/MS Conditions for all analytes**.

**MS Condition**	**Cor1-C420**	**Cysteine-containing heptapeptide**	**Lysine-containing heptapeptide**	**α-N-acetyl leucine enkephalin**	**Leucine enkephalin acetate salt hydrate**
Collision-induced dissociation (CAD) gas	9	5	5		
Curtain gas (CUR)	40	30	30		
Nebuliser	65	55	55		
Ion spray temperature (TEM)	550	550	550		
Collision energy (CE)	45	95	27	63	71
Collision cell exit potential (CXP)	4	4	4	4	4
Declustering potential (DP)	41	111	51	51	51
Entrance potential (EP)	7	11.5	9	9.5	9.5
MS/MS transition	455.3 → 120.0	751.3 → 120.0	389.0 → 129.3	598.4 → 120.1	556.2 → 120.1

#### Preparation of peptide standards, calibration curves, quality control (QC) samples, and test compounds with known sensitizing capacity

An eight-point calibration curve for each heptapeptide (Cor1-C420, 5–50 μM; cysteine-containing heptapeptide, 2–100 μM; lysine-containing heptapeptide, 2–100μM) was prepared. Duplicates of three standard QC samples [three times the lower limit of quantitation (LLOQ), 50% of the upper limit of quantitation (ULOQ), 80% of the ULOQ] were prepared in 0.1 M phosphate buffer (pH7.4) for Cor1-C420 and the heptapeptide containing cysteine, whereas 0.1 M ammonium acetate buffer (pH10) was used for the heptapeptide containing lysine. The QC concentrations for Cor1-C420 were 15, 25, and 40 μM whereas the QC concentrations for the heptapeptides containing cysteine and lysine were 6, 50, and 80 μM. Test chemicals with known sensitizing capacity were used as total peptide depletion controls. DNCB (extreme sensitizer), isoeugenol (moderate sensitizer), cinnamaldehyde (moderate sensitizer), and methyl salicylic acid (non-sensitizer) were used to assess the stability of the Cor1-C420 and cysteine-containing heptapeptide complexes after their formation. For the lysine-containing heptapeptide, glutaraldehyde (strong sensitizer), and ethyl acrylate (weak sensitizer) were used in place of DNCB and isoeugenol. These chemicals were prepared in acetonitrile; the final percentage of organic solvent (acetonitrile) did not exceed 27% in the buffer solution. The final reaction volume was 300 μL. The molar ratio of the Cor1-C420 and cysteine-containing heptapeptides to test chemical in the incubation mixtures was 1:10. By comparison the corresponding ratio for the lysine-containing heptapeptide and the test chemicals was 1:50.

#### Peptide reactivity assessment

After 24 h of incubation, leucine enkephalin acetate salt hydrate (75 μL, 12 μg/mL) or α-N-acetyl leucine enkephalin (75 uL, 100 μg/mL) as internal standard, was added to the samples prior to a 1 in 20 dilution for the cysteine- and lysine-containing heptapeptides, and a 1 in 8 dilution for Cor1-C420 in 5% acetonitrile in water prior to final analysis. For the cysteine-containing heptapeptide, an additional step was needed to prevent dimerization of the thiol groups. Specifically, 10 μL aliquots of 16 mM DTT were added to each diluted sample (final volume 200 μL) followed by incubation for 30 min at 40°C.

#### Carry-over assessment and lower limit of quantification (LLOQ)

The LLOQ was assessed using the criteria that the analyte response at the LLOQ must be five times the baseline noise and it should have an accuracy of ±20% of the nominal concentration (EMA, 2011). The carry-over was assessed by injecting the highest concentration, the upper limit of quantification (ULOQ) of the analyte followed by a “blank” sample that did not contain the analyte of interest, but in the same buffer. The carry-over should not be more than 20% of the LLOQ (EMA, 2011).

#### Incubation temperature stability

Calibration curve for all three tested heptapeptides ranging from 2 to 50 μM were incubated at each of three temperatures, viz 4, 25, or 37°C for a time period of 24 (±1) h. The peptide concentrations were then assessed as per the methods described in Section Peptide Reactivity Assessment.

#### Adsorption of heptapeptides on polypropylene and glass materials

To assess the extent to which there were adsorptive losses of each of the three heptapeptides of interest onto the vial materials over time, standard calibration curves, standard QC samples, and four test chemical control samples as described in Section Preparation of Peptide Standards, Calibration Curves, Quality Control (QC) Samples, and Test Compounds with Known Sensitizing Capacity, were prepared in both 96-well polypropylene plates and borosilicate glass vials throughout the course of experiment. These samples were incubated at 25°C for a period of 24 (±1) h and were placed in the autosampler and injected once every 24 h for a 3 day period. The calculated concentrations on days 1, 2, and 3 were compared with that determined on day 0. Accuracy and precision were calculated from the duplicates of the QC samples included in each experiment and three independent experiments were performed. Thus, we had a total of six values for each QC at each concentration (low, medium, and high).

$$\text{Accuracy}=\frac{\begin{array}{l} Nominalconcentration\\ -\;Mean\;of\;the\;calculated\;concentration \end{array}}{Nominal\;concentration}\times 100\%$$

$$\text{Precision}=\frac{Standard\;deviation\;of\;calculated\;concentration}{Mean\;of\;the\;calculated\;concentration}\times 100\%$$

#### Stability of the peptide-chemical complexes when stored in autosampler

Standard calibration curves, QC samples, and test chemical control samples with known sensitizing capacity were prepared as per the description in Section Preparation of Peptide Standards, Calibration Curves, Quality Control (QC) Samples, and Test Compounds with Known Sensitizing Capacity. After a mean (±SD) incubation period of 24 (±1) h at 25°C, the standard calibration curve samples, standard QC samples, and test chemical control samples were placed in the autosampler at 4°C and the stability of the heptapeptides was monitored for 3 days post-incubation. The back-calculated concentration of the calibration standards should be within ±15% of the nominal value, except for the LLOQ for which it should be within ±20% (EMA, 2011). At least 75% of the calibration standards must fulfill these acceptance criteria for assay validation. QC sample accuracy should be within ±15% of the nominal values. At least 67% of the QC samples should comply with these criteria. If any of these criteria was not met, then the analytical batch was rejected.

#### Linearity

Calibration curve linearity was assessed on three independent experiments. A linear least squares regression model with 1/x weighting was applied to all calibration curves. The assay range was considered linear when the back calculated concentrations and the coefficient of variation (CV) of the calibration standards were within ±15% of the nominal concentrations, except for the LLOQ for which ±20% was acceptable. The same criteria were applied to the peptide depletion response by the reference control (i.e., 50 μM Cor1-C420 and 100 μM cysteine or lysine containing heptapeptides).

#### Data analysis

The percent heptapeptide depletion was calculated using Equation 1. Our findings were compared with the OECD TG442C for reactivity classification and DPRA prediction (OECD, 2015). The total depletion of Cor1-C420 and lysine heptapeptide were compared against the values in Table 2 as Cor1-C420 contains both cysteine and lysine side chains. The total cysteine-containing heptapeptide depletion was compared against the values in Table 3.

(1) $$\begin{array}{l} \%\text{ Depletion}=\frac{\left(\begin{array}{c} \text{Mean peptide}\\ \text{concentrationin}\\ \text{the absence of}\\ \text{test chemical} \end{array}\right)-\left(\begin{array}{c} \text{Mean peptide}\\ \text{concentration in}\\ \text{the presence of}\\ \text{test chemical} \end{array}\right)}{\left(\begin{array}{c} \text{Mean peptide concentration in}\\ \text{the absence of the test chemical} \end{array}\right)}\\ \;\times \;100\% \end{array}$$

**Table 2:** **Percent peptide depletion model based upon cysteine 1:10 and lysine 1:50 (OECD, 2015)**.

**Mean of cysteine and lysine % depletion**	**Reactivity class**	**DPRA prediction**
0% ≤ mean % depletion ≤ 6.38%	No/minimal reactivity	Negative
6.38% < mean % depletion ≤ 22.62%	Low reactivity	Positive
22.62% < mean % depletion ≤ 42.47%	Moderate reactivity	
42.47% < mean % depletion ≤ 100%	High reactivity	

**Table 3:** **Percent peptide depletion model based upon cysteine 1:10 (OECD, 2015)**.

**Cysteine % depletion**	**Reactivity class**	**DPRA prediction**
0% ≤ % depletion ≤ 13.89%	No/minimal reactivity	Negative
13.89% < mean % depletion ≤ 23.09%	Low reactivity	Positive
23.09% < mean % depletion ≤ 98.24%	Moderate reactivity	
98.24% < mean % depletion ≤ 100%	High reactivity	

## Results

### Chromatography

**Figure 2:**
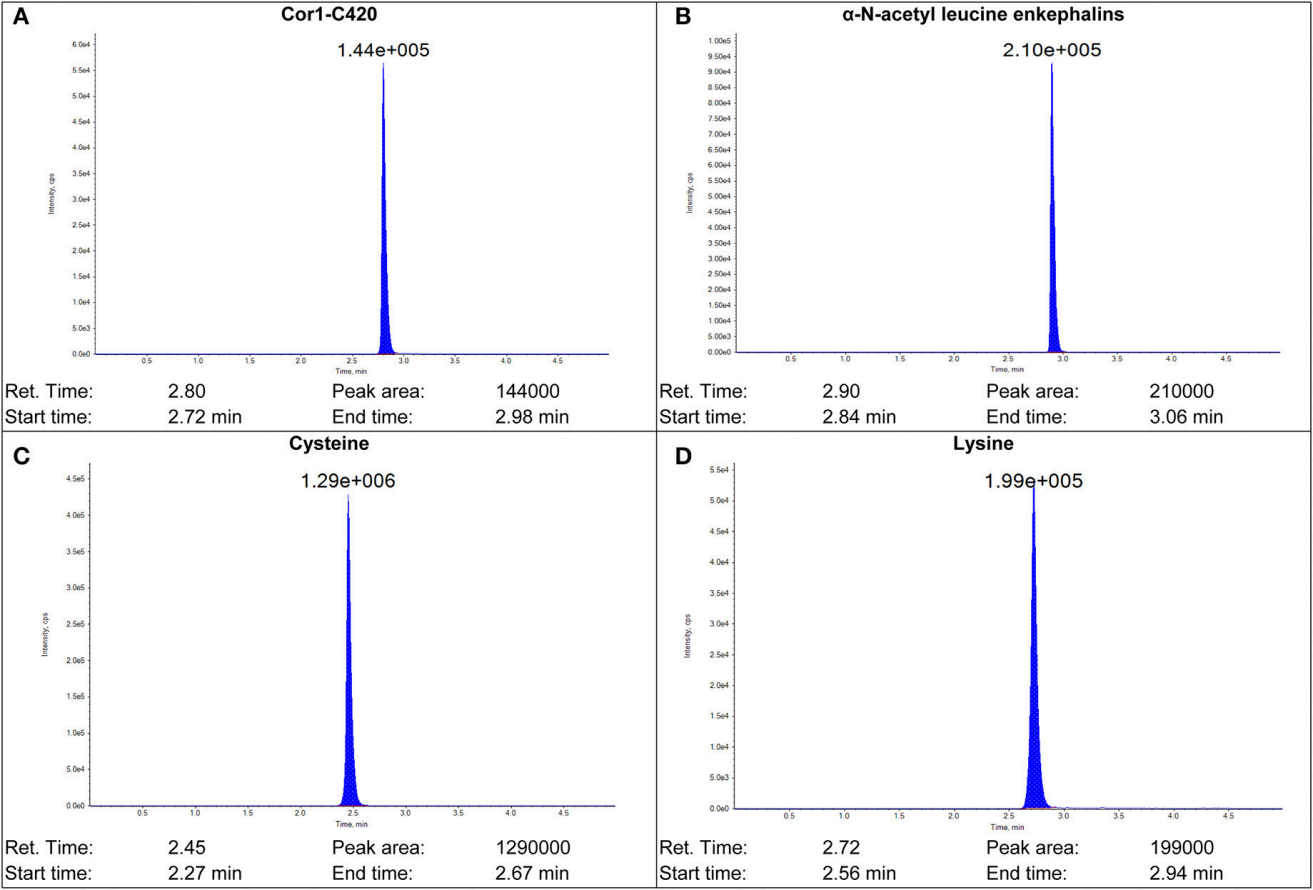
**Sample chromatograms of (A) Cor1-C420 (B) internal standard α-N-acetyl leucine enkephalin (C) cysteine- and (D) lysine-containing heptapeptides**.

The MS/MS transitions and optimized MS parameters as well as the chromatograms of the peptides and internal standards are presented in Table 1 and Figure 2, respectively.

### Carry-over assessment and LLOQ

The percent carry-over was calculated using Equation 2. Carry-over was observed for Cor1-C420, such that the peak area of the heptapeptide detected in the blank was 10% of the response from the LLOQ. However, this carry-over was within the acceptance criteria of not more than 20% of the LLOQ peak area. No carry-over was observed for cysteine- and lysine-containing heptapeptides or for the internal standard. The LLOQ for Cor1-C420 was 5 μM whereas the LLOQ for both the cysteine- and lysine-containing heptapeptides was 2 μM.

(2) $$\begin{array}{l} \text{Carry}-\text{over }(\%)=\frac{\text{Analyte area detected in the blank sample}}{\text{Analyte area of LLOQ}}\\ \;\times \text{ 100}\% \end{array}$$

### Incubation temperature stability

Statistical analysis was performed using repeated measures two-way analysis of variance (ANOVA) followed by the Bonferroni test to assess the stability of heptapeptides between incubation temperatures. Statistical analysis was carried out using the GraphPad Prism™ software program (Version 6.04) and the statistical significance criterion was *p* < 0.05.

All the standard calibration curves were compared with freshly prepared standard calibration curve. The standard calibration curves for Cor1-C420 that was incubated at 25 and 37°C were significantly different (*P* < 0.05 and *P* < 0.0001, respectively), from that for the freshly prepared standard calibration curve. This could be due to instability of Cor1-C420 at ambient or high temperatures. No significance difference was observed for the peptide standards that were incubated at 4°C for a period of 24 (±1) h (*P* > 0.05). By contrast, the cysteine- and lysine-containing heptapeptides remained stable for 24 h at 4, 25, and 37°C with no significant difference (*P* > 0.05) observed for each peptide at the various incubation temperatures.

### Adsorption of heptapeptides onto polypropylene and borosilicate glass vessels

Peptide stability was assessed for Cor1-C420 (at 15, 25, and 40 μM) and for the heptapeptides containing lysine or cysteine (at 6, 50, and 80 μM) over 3 days in vessels made of polypropylene and borosilicate glass materials (Table 4). Our data show that the Cor1-C420 concentration for QCs samples prepared at low, medium and high concentrations remained unchanged in polypropylene vials. The accuracy of all three Cor1-C420 QC samples across 3 days was within ±15% of their respective nominal concentrations. By contrast, all Cor1-C420 analytical batches incubated in borosilicate glass vials were rejected for days 1–3 as the repeated analyses did not meet the acceptance criteria as specified in Section Linearity. The Cor1-C420 standard curve failed the linearity assessment and hence the accuracy of the QC samples for this peptide (in glass) was not determined.

**Table 4:** **Summary of precision and accuracy in percentage for QC samples of all three heptapeptide in 96-well polypropylene plate assessed at 24 h intervals for Days 0–3**.

**Heptapeptides**		**Day 0 (*****n*** = 3**)**			**Day 1 (*****n*** = 3**)**			**Day 2 (*****n*** = 3**)**			**Day 3 (*****n*** = 3**)**		
		**Low**	**Med**	**High**	**Low**	**Med**	**High**	**Low**	**Med**	**High**	**Low**	**Med**	**High**
Cor1-C420	Precision (%)	6.3	6.9	8.5	6.1	7.4	4.7	6.6	11.0	13.1	11.8	13.3	10.4
	Accuracy (%)	1.2	4.5	−2.6	0.1	2.4	−4.6	1.6	4.3	−3.0	−1.5	3.1	−0.6
Cysteine-containing heptapeptide	Precision (%)	6.7	5.5	4.6	7.4	7.6	4.5	4.9	3.6	5.4	7.4	3.5	5.6
	Accuracy (%)	−7.3	−4.4	−2.5	6.4	0.3	0.1	−0.7	0.2	−3.0	−4.4	−1.4	−2.3
Lysine−containing heptapeptide	Precision (%)	5.9	3.4	6.0	6.0	1.5	6.4	3.7	2.1	9.0	4.8	1.6	11.7
	Accuracy (%)	−7.4	−0.4	−4.4	−4.4	−2.4	−5.7	−6.8	−2.0	−6.1	−5.4	−1.6	−7.3

The accuracy of QC samples was within the acceptance criterion, i.e., ±15% from the nominal concentration.

The concentration of the cysteine-containing heptapeptide QC samples remained unchanged when stored in vessels made from polypropylene materials throughout the course of the experiment (Table 4). Additionally, the cysteine-containing heptapeptide QC samples remained unchanged for up to 2 days post incubation in borosilicate glass vials. However, the cysteine-containing heptapeptide standard curve failed the linearity assessment on day 3. As for the lysine-containing heptapeptide, the standard curves remained unchanged in vessels made from both polypropylene (Table 4) and borosilicate glass for up to 3 days post-incubation.

### Stability of the peptide-chemical complexes when stored in autosampler

The stability of the peptide-chemical complexes stored in HPLC autosampler plates was assessed using chemicals with known sensitizing capacities, viz DNCB, isoeugenol, cinnamaldehyde, and methyl salicylate, with Cor1-C420 and the heptapeptide containing cysteine (Tables 5, 6). The corresponding data for glutaraldehyde, cinnamaldehyde, ethyl acrylate, and methyl salicylate incubated with the heptapeptide containing lysine are shown in Table 7. The autosampler stability of the peptide-chemical complexes was determined to assess the feasibility of injecting a large number of samples in a single analytical experiment without adversely affecting sample integrity which would be a requirement for conducting the peptide reactivity assay in high-throughput format.

**Table 5:** **Percent depletion of the Cor1-C420 heptapeptide incubated with representative test chemicals in polypropylene and borosilicate glass vessels for a period of 24 (±1) h post-incubation (*n* = 3)**.

**Test chemicals**	**Day post incubation**	**Polypropylene vessel**		**Borosilicate glass vessel**	
		**Mean % depletion (±SD)**	**Classification of test chemical^1^**	**Mean % depletion (±SD)**	**Classification of test chemical^1^**
DNCB (Strong sensitiser)	0	97.14 (±1.0)	High reactivity	98.18 (±1.6)	High reactivity
	1	93.78 (±0.7)	High reactivity	N/A	
	2	92.86 (±1.5)	High reactivity	N/A	
	3	92.56 (±3.4)	High reactivity	N/A	
Isoeugenol (Moderate sensitiser)	0	64.08 (±1.2)	High reactivity	70.07 (±5.5)	High reactivity
	1	72.96 (±2.1)	High reactivity	N/A	
	2	80.37 (±6.0)	High reactivity	N/A	
	3	82.07 (±5.0)	High reactivity	N/A	
Cinnamaldehyde (Moderate sensitiser)	0	33.83 (±8.1)	Moderate reactivity	35.76 (±5.8)	Moderate reactivity
	1	17.66 (±12.7)	Low reactivity^*^	N/A	
	2	10.47 (±10.9)	No/minimal Reactivity^*^	N/A	
	3	5.21 (±6.3)	No/minimal Reactivity^*^	N/A	
Methyl salicylate (Weak sensitiser)	0	7.54 (±7.1)	No reactivity	8.84 (±5.4)	Low reactivity
	1	11.24 (±8.0)	Low reactivity^*^	N/A	
	2	14.56 (±9.4)	Low reactivity^*^	N/A	
	3	19.18 (±10.7)	Low reactivity^*^	N/A	

*Day 0 in the table denotes the first day of sample storage in an autosampler at 4°C. The mean depletion is calculated based on the data from three replicates from each of three independent experiments*.

* *change in reactivity class; N/A denotes the batch failed acceptance criteria*.

**Table 6:** **Percent depletion of the cysteine heptapeptide incubated with representative test chemicals in polypropylene and borosilicate glass vessels for a period of 24 h (±1) h post incubation (*n* = 3)**.

**Test chemicals**	**Day post incubation**	**Polypropylene vessel**		**Borosilicate glass vessel**	
		**Mean % depletion (±SD)**	**Classification of test chemical^2^**	**Mean % depletion (±SD)**	**Classification of test chemical^2^**
DNCB (Strong sensitiser)	0	88.74 (±2.5)	Moderate reactivity	85.13 (±1.7)	Moderate reactivity
	1	81.36 (±1.0)	Moderate reactivity	73.83 (±2.6)	Moderate reactivity
	2	75.71 (±1.6)	Moderate reactivity	64.09 (±2.7)	Moderate reactivity
	3	75.83 (±0.9)	Moderate reactivity	N/A	
Isoeugenol (Moderate sensitiser)	0	32.84 (±7.0)	Moderate reactivity	38.77 (±6.9)	Moderate reactivity
	1	29.41 (±3.9)	Moderate reactivity	35.90 (±0.5)	Moderate reactivity
	2	24.54 (±6.2)	Moderate reactivity	30.99 (±2.5)	Moderate reactivity
	3	28.73 (±7.0)	Moderate reactivity	N/A	
Cinnamaldehyde (Moderate sensitiser)	0	27.40 (±2.9)	Moderate reactivity	35.26 (±2.3)	Moderate reactivity
	1	22.65 (±0.5)	Low reactivity^*^	24.34 (±7.5)	Moderate reactivity
	2	21.10 (±2.8)	Low reactivity^*^	16.82 (±6.7)	Low reactivity^*^
	3	22.66 (±4.0)	Low reactivity^*^	N/A	
Methyl salicylate (Weak sensitiser)	0	0.50 (±0.9)	No/minimal reactivity	5.94 (±3.3)	No/minimal reactivity
	1	0.00 (±0.0)	No/minimal reactivity	0.45 (±4.5)	No/minimal reactivity
	2	0.95 (±1.6)	No/minimal reactivity	1.72 (±0.9)	No/minimal reactivity
	3	0.84 (±1.5)	No/minimal reactivity	N/A	

*Day 0 in the table denotes the first day of sample storage in an autosampler at 4°C. The mean depletion is calculated based on the data from three replicates from each of three independent experiments*.

* *change in reactivity class; N/A denotes the batch failed acceptance criteria*.

**Table 7:** **Percent depletion of the lysine heptapeptide incubated with representative test chemicals in polypropylene and borosilicate glass vessels for a period of 24 h (±1) h post incubation (*n* = 3)**.

**Test chemicals**	**Day post incubation**	**Polypropylene vessel**		**Borosilicate glass vessel**	
		**Mean % depletion (±SD)**	**Classification of test chemical^3^**	**Mean % depletion (±SD)**	**Classification of test chemical^3^**
Glutaraldehyde (Strong sensitiser)	0	49.23 (±5.8)	High reactivity	55.33 (±4.0)	High reactivity
	1	49.03 (±5.2)	High reactivity	58.68 (±3.7)	High reactivity
	2	49.61 (±5.3)	High reactivity	61.98 (±4.8)	High reactivity
	3	51.25 (±6.2)	High reactivity	66.51 (±7.4)	High reactivity
Cinnamaldehyde (Moderate sensitiser)	0	7.18 (±6.7)	Low Reactivity	9.89 (±6.1)	Low Reactivity
	1	4.89 (±5.2)	No/minimal Reactivity^*^	6.28 (±2.4)	No/minimal Reactivity^*^
	2	4.36 (±4.7)	No/minimal Reactivity^*^	4.88 (±1.7)	No/minimal Reactivity^*^
	3	4.08 (±4.6)	No/minimal Reactivity^*^	4.40 (±0.8)	No/minimal Reactivity^*^
Ethyl acrylate (Weak sensitiser)	0	24.73 (±9.8)	Moderate reactivity	47.28 (±7.7)	High reactivity
	1	23.54 (±7.7)	Moderate reactivity	43.40 (±5.9)	High reactivity
	2	23.68 (±6.4)	Moderate reactivity	42.05 (±6.1)	Moderate reactivity^*^
	3	23.42 (±6.6)	Moderate reactivity	42.18 (± 6.2)	Moderate reactivity^*^
Methyl salicylate (Non-sensitiser)	0	9.14 (±9.4)	Low Reactivity	3.52 (±5.5)	No/minimal Reactivity
	1	8.02 (±7.6)	Low Reactivity	2.89 (±1.7)	No/minimal Reactivity
	2	7.72 (±6.5)	Low Reactivity	1.52 (±0.4)	No/minimal Reactivity
	3	7.94 (±6.6)	Low Reactivity	1.90 (±0.7)	No/minimal Reactivity

*Day 0 in the table denotes the first day of sample storage in an autosampler at 4°C. The mean depletion is calculated based on the data from three replicates from each of three independent experiments*.

* *change in reactivity class*.

Stability of the peptide-chemical complexes was assessed in polypropylene plates for the Cor1-C420 and cysteine-containing heptapeptides due to the significant losses of both peptides onto glass materials as reported in Section Adsorption of Heptapeptides onto Polypropylene and Borosilicate Glass Vessels. The total peptide depletion of chemicals with known sensitizing potential for days 1–3 was compared against those determined on day 0. Following incubation of each of DNCB and cinnamaldehyde with Cor1-C420, there was a decrease in percent peptide depletion over the 3-day assessment period (i.e., an increase in peptide concentration). Importantly, this was not extensive and so the classification of these chemicals with respect to reactivity class did not change. However, following incubation of isoeugenol and methyl salicylate with Cor1-C420, the reverse trend was observed such that there was a marked increase in peptide depletion over the 3-day assessment period (Table 5), that would lead to eventual misclassification of the reactivity of each of these chemicals. For example, cinnamaldehyde was initially assessed as having moderate peptide reactivity when assessed on day 0 which was in line with known LLNA data, with the reactivity gradually decreasing with minimal/no reactivity by 48 h post-chemical incubation (day 1).

Test chemicals incubated with cysteine-containing heptapeptides showed a decrease in peptide depletion over the 3-day assessment period (i.e., an increase in peptide concentration) for DNCB, isoeugenol, and cinnamaldehyde (Table 6). In particular, the change in peptide depletion over time resulted in cinnamaldehyde initially being categorized as having moderate reactivity on day 0 but with this changing to low reactivity from day 1 onwards.

As there were no losses of the lysine-containing heptapetide in glass or polypropylene vessels (Section Adsorption of Heptapeptides onto Polypropylene and Borosilicate Glass Vessels), the stability of the formed peptide-chemical complexes for the test chemicals, glutaraldehyde, cinnamaldehyde, ethyl acrylate, and methyl salicylate were assessed using vessels made from both types of materials. With the exception of cinnamaldehyde, the extent of lysine-containing heptapeptide depletion over the 3-day assessment period remained unchanged for glutaraldehyde, ethyl acrylate, and methyl salicylate in reactions carried out in polypropylene plates (Table 7). However, the total lysine-containing heptapeptide depletion by ethyl acrylate (weak sensitizer) was ~20% higher overall for the entire 3-day assessment period when the reaction was carried out in borosilicate glass vials compared with the corresponding data generated using polypropylene vials (Table 7). This apparent difference in the extent of lysine-containing heptapetide depletion between reactions carried out in polypropylene vs. borosilicate glass vials was not evident for glutaraldehyde, cinnamaldehyde, and methyl salicylate as the total lysine-containing heptapetide depletion was similar (±15%) for reactions conducted in both polypropylene and borosilicate glass vials.

Overall our present data indicate that the stability of the covalent bonds formed between the test chemical and heptapeptide of interest, appears to be dependent upon the type of chemical being assessed as well as the heptapeptides utilized. Although the number of test chemicals assessed was small, our data suggest that the total elapsed time for conduct of the peptide reactivity assay irrespective of the heptapeptide used, should not exceed 24 h in order to maximize assay accuracy which is in line with OECD TG442C (OECD, 2015).

### Linearity

Calibration curves were linear and the slope, y-intercept and regression coefficient (*R*^2^) were determined. Data showing calibration curve linearity for all three heptapeptides using polypropylene vials are summarized in Tables 8–10. Our calibration data showed high precision (<10%) and high accuracy (<10%) between each replicate and days of the assay. The mean slope for Cor1-C420, cysteine,- and lysine-containing heptapeptides was 0.0351, 0.0521, and 0.0306 while the mean *R*^2^ values were 0.9876, 0.9951, and 0.9958, respectively.

**Table 8:** **Calibration curve linearity for the Cor1-C420 heptapeptide (*n* = 3) in 96-well polypropylene plate over 3 days**.

**Nominal**	**Mean measured concentration (C**_**m**_**)**												**Mean**	**SD**	**Precision**	**Accuracy**
**Conc. (μM)**	**Replicate 1**				**Replicate 2**				**Replicate 3**						**(%)**	**(%)**
	**Day 0**	**Day 1**	**Day 2**	**Day 3**	**Day 0**	**Day 1**	**Day 2**	**Day 3**	**Day 0**	**Day 1**	**Day 2**	**Day 3**				
5	4.72	5.10	5.20	5.16	4.66	5.09	4.94	5.42	4.96	5.24	5.61	5.77	5.16	0.33	6.4	3.1
10	9.65	9.74	9.43	9.50	10.0	9.91	9.70	9.14	10.6	10.0^*^	8.93	8.59^*^	9.61	0.55	5.7	−3.9
15	15.4	14.9	14.9	15.6	15.1	14.6	14.2	14.4	14.3	14.2	15.1	14.8	14.78	0.46	3.1	−1.4
20	20.1	19.8	19.5	20.4	20.5	19.8	21.5	20.4	18.7	21.6	20.0	19.3	20.13	0.83	4.1	0.7
25	27.0	25.5	25.9	23.4^*^	26.1	25.2	26.0	25.8	25.8	22.8	22.8	21.9	24.85	1.67	6.7	−0.6
30	31.4	29.5	30.6	28.7^*^	30.9	30.9	29.9	29.0^*^	30.8	30.5^*^	30.8	29.9	30.24	0.85	2.8	0.8
40	39.4	41.7	40.8	37.7^*^	40.8	40.0	41.7	40.6	40.6	41.8	41.0	43.0	40.76	1.33	3.3	1.9
50	47.3	48.7	48.7	51.9	46.9	49.5	47.1	49.8	49.2	49.8	50.8	51.1	49.25	1.60	3.3	−1.5
A	0.0580	0.0465	0.0325	0.0336	0.0483	0.0446	0.0453	0.0453	0.0234	0.0165	0.0139	0.0138	0.0351			
B	−0.0546	−0.1400	−0.1220	−0.1490	−0.0330	−0.1250	−0.1640	−0.2050	−0.0344	−0.0383	−0.0492	−0.0597	−0.0979			
*R*^2^	0.9800	0.9972	0.9830	0.9762	0.9938	0.9980	0.9889	0.9835	0.9881	0.9878	0.9907	0.9839	0.9876			

* *denotes single data point was used*.

**Table 9:** **Calibration curve linearity of cysteine heptapeptide (*n* = 3) in 96-well polypropylene plate over 3 days**.

**Nominal**	**Mean measured concentration (C**_**m**_**)**												**Mean**	**SD**	**Precision**	**Accuracy**
**Conc. (μM)**	**Replicate 1**				**Replicate 2**				**Replicate 3**						**(%)**	**(%)**
	**Day 0**	**Day 1**	**Day 2**	**Day 3**	**Day 0**	**Day 1**	**Day 2**	**Day 3**	**Day 0**	**Day 1**	**Day 2**	**Day 3**				
2	2.34	2.11^*^	N/A	2.15	1.84	1.81	N/A	2.03	N/A	N/A	1.87	N/A	2.02	0.19	9.6	1.1
5	4.78	4.76^*^	N/A	4.82	4.90	4.89	N/A	4.99	N/A	5.56^*^	4.97	N/A	4.96	0.26	5.2	−0.8
10	9.29	9.06^*^	8.98	9.62	10.5	10.4	8.91	9.64	10.9	9.99	10.1	10.9	9.85	0.71	7.2	−1.5
20	18.0	20.4	20.5	19.2	20.7	21.1	21.2	20.3	19.0	18.9	20.6	18.6	19.88	1.07	5.4	−0.6
30	30.2	29.4^*^	32.1	30.6	30.8	31.0	31.8	30.5	28.5	29.4	30.7	28.6	30.30	1.14	3.8	1.0
50	51.2	52.7	52.3	50.5	51.2	51.3	51.3	49.7	49.6	50.5	52.2	50.7	51.10	0.98	1.9	2.2
80	80.8	80.5	79.0	81.0	78.7	78.2	78.0	79.9	81.2	80.3	79.5	80.9	79.85	1.13	1.4	−0.2
100	100.4	97.2	97.0	99.0	98.4	98.2	98.7	99.9	101	101	97.1	100	99.06	1.55	1.6	−0.9
A	0.0291	0.0429	0.0529	0.0376	0.0432	0.0442	0.0547	0.0351	0.0489	0.0755	0.1030	0.0577	0.0521			
B	−0.0255	−0.0217	0.1920	−0.0198	0.0165	0.0262	0.2060	0.0031	−0.2070	−0.1580	0.0116	−0.2620	−0.0199			
*R*^2^	0.9981	0.9903	0.9919	0.9979	0.9968	0.9957	0.9950	0.9993	0.9954	0.9942	0.9944	0.9922	0.9951			

* *denotes single data point was used; N/A denotes the points were excluded*.

**Table 10:** **Calibration curve linearity of lysine heptapeptide (*n* = 3) in 96-well polypropylene plate over 3 days**.

**Nominal**	**Mean measured concentration (C**_**m**_**)**												**Mean**	**SD**	**Precision**	**Accuracy**
**Conc. (μM)**	**Replicate 1**				**Replicate 2**				**Replicate 3**						**(%)**	**(%)**
	**Day 0**	**Day 1**	**Day 2**	**Day 3**	**Day 0**	**Day 1**	**Day 2**	**Day 3**	**Day 0**	**Day 1**	**Day 2**	**Day 3**				
2	1.96	1.93	1.92	1.85	1.87	1.95	1.96	1.97	1.84	1.92	1.86	1.92	1.91	0.05	2.5	−4.4
5	4.94	5.08	4.98	4.98	4.92	4.92	4.86	4.80	4.98	4.96	4.94	5.00	4.95	0.07	1.4	−1.1
10	9.89	10.0	9.93	9.98	10.4	10.4	10.2	10.1	10.2	10.0	10.3	9.99	10.13	0.18	1.7	1.3
20	20.4	20.4	20.8	21.1	20.9	20.4	20.6	21.0	20.8	20.5	20.8	20.3	20.67	0.27	1.3	3.3
30	30.7	29.8	30.6	31.4	31.0	30.4	30.7	30.8	31.0	30.8	30.8	31.0	30.74	0.38	1.2	2.5
50	50.7	50.3	50.7	50.9	49.3	48.8	49.2	48.4	51.0	50.6	50.2	51.0	50.09	0.92	1.8	0.2
80	79.9	79.4	78.7	78.4	77.2	76.5	77.5	78.9	78.7	79.5	79.7	79.5	78.65	1.08	1.4	−1.7
100	98.6	100	99.4	98.4	101.5	104	102	101	98.4	98.6	98.4	98.4	99.87	1.77	1.8	−0.1
A	0.0367	0.0332	0.0379	0.0378	0.0251	0.0257	0.0293	0.0302	0.0264	0.0279	0.0289	0.0280	0.0306			
B	0.0037	0.0016	0.0075	0.0098	0.0067	0.0035	0.0013	0.0033	0.0065	0.0043	0.0036	0.0007	0.0044			
*R*^2^	0.9845	0.9984	0.9990	0.9973	0.9952	0.9968	0.9977	0.9976	0.9958	0.9950	0.9962	0.9959	0.9958			

## Discussion

We used a comprehensive and systematic approach to identify the optimal experimental conditions for conducting the peptide reactivity assay in 96-well plate format with LC-MS/MS quantification of the extent of peptide depletion. Specifically, the optimal assay incubation temperature was 25°C for the three heptapeptides assessed (Cor1-C420, heptapeptides containing cysteine and lysine), as incubation at 37°C adversely affected Cor1-C420 peptide stability.

### Heptapeptides on polypropylene and glass materials

Our data comparing the effects of using a 96-well polypropylene plate relative to borosilicate glass vials on losses of heptapeptides as well as on the stability of peptide-chemical complexes is an extension studies described by Natsch and Gfeller (2008), Roberts and Natsch (2009), and Roberts and Aptula (2014). Importantly, we found that polypropylene plates were preferable to glass vials in terms of minimizing losses of the peptides of interest even though glass vials are more commonly used for heptapeptide reactivity assessments. Our findings extend the existing peptide reactivity assay especially for the example of the heptapeptide containing lysine-ethyl acrylate complex where total lysine-containing heptapeptide depletion was ~20% lower when the assay was conducted in polypropylene compared with glass vials under the same assay preparation conditions. Furthermore, our findings show that the peptide reactivity assay may not be suitable for screening a large number of chemicals in a single experiment due to the potential for instability of test chemical-peptide complexes such that the peptide concentration may change significantly when stored in an autosampler over a 3-day period.

Next, we assessed the impact of the reaction vial materials (polypropylene or borosilicate glass) used for test chemical incubation reactions on apparent peptide depletion. Our data clearly show that the Cor1-C420 and cysteine-containing heptpeptides were less affected by polypropylene than by borosilicate glass. Specifically, the Cor1-C420 and cysteine-containing heptapeptide QCs did not pass the acceptance criteria for samples processed in glass vials after autosampler storage at 4°C for periods of 24 h (day 1) and 72 h (day 3), respectively, in contrast to similar samples processed in polypropylene plates where the QC samples passed the assay acceptance criteria. The use of either polypropylene or glass materials for the incubation step did not appear to cause non-specific adsorptive losses of lysine-containing peptide, with the concentrations of all QC samples within the acceptance criterion of ±15% of the nominal peptide concentrations. However, incubation of ethyl acrylate (weak sensitizer) with lysine-containing heptapeptide in glass or polypropylene materials showed that the apparent total lysine-containing heptapeptide depletion was 47.3 or 24.7%, respectively, when assessed within 24 h of test chemical addition to the peptide. However, in work by others, ethyl acrylate gave different percentages of lysine-containing heptapeptide depletion, at 2.1 and 93.7% (Gerberick et al., 2007; Troutman et al., 2011), results that would misclassify ethyl acrylate as having no/minimal or strong reactivity, respectively. The varying reports on total lysine-containing heptapeptide depletion with ethyl acrylate could be due to different experimental conditions employed in each case.

### Incubation temperature stability

**Figure 3:**
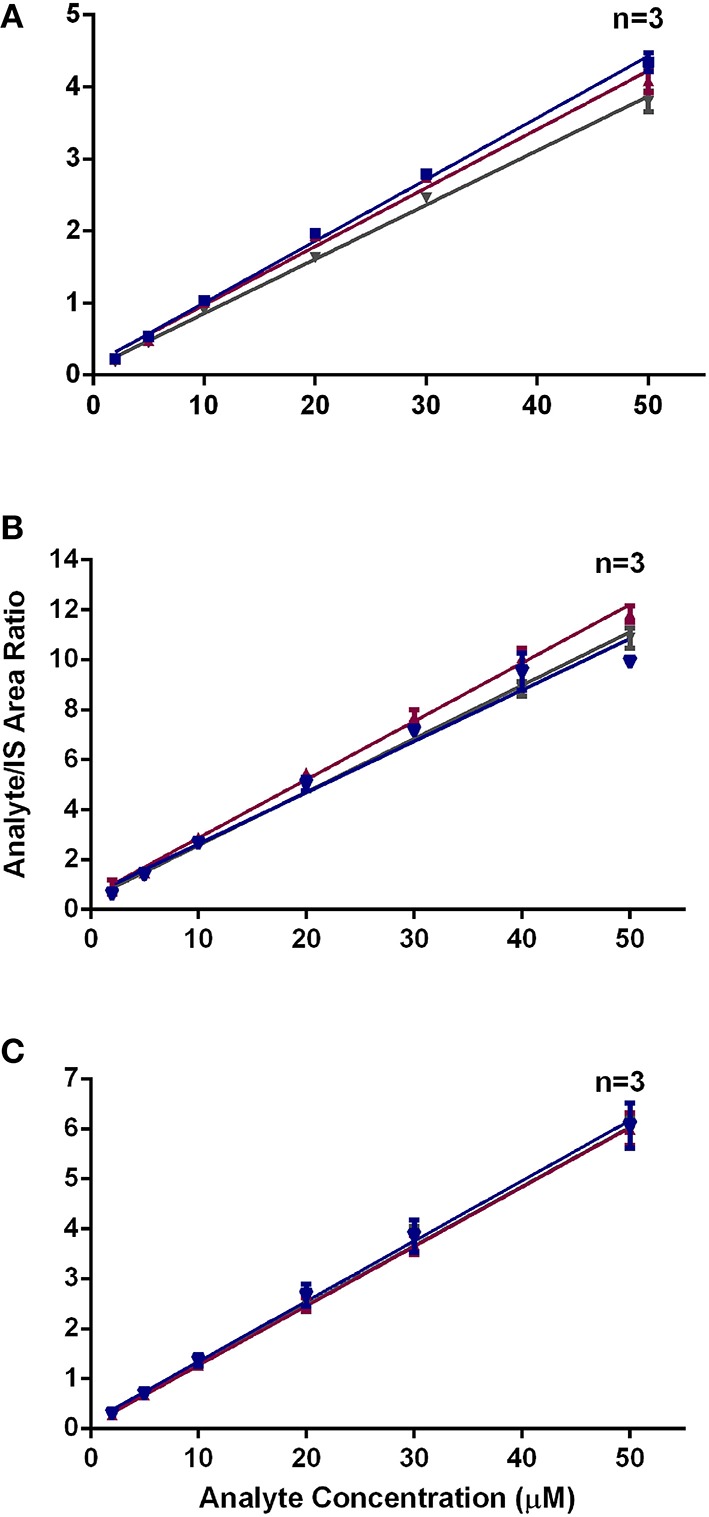
**Area ratio of (A) Cor1-C420 (B) cysteine- and (C) lysine-containing heptapeptides with respect to internal standard at different incubation temperature**.

Our present findings on the effects of varying the incubation temperature employed in the peptide reactivity assay, mimicking the various temperatures used by laboratories globally, on the stability of the heptapeptides, are also novel. Natsch and Gfeller (2008) used 37°C for incubating various test chemicals with the Cor1-C420 heptapeptide, whereas Gerberick et al. (2007) and the OECD guideline, TG442C, recommend a 24 h incubation period at a temperature of 25°C for test chemicals with cysteine- and lysine-containing heptapeptides (OECD, 2015). Herein, we compared the effect of these two incubation temperatures (25 and 37°C) for representative test chemicals with a range of concentrations of all three heptapeptides, viz, Cor1-C420, cysteine- and lysine-containing heptapeptides with that of freshly prepared samples as the control condition. Our findings show that an incubation temperature of 37°C may induce loss of Cor1-C420 (Figure 3). By comparison, a temperature of 4°C did not significantly alter the stability of these three heptapeptides. A temperature of 25°C was selected as the optimal temperature for subsequent reactions of test chemicals with each of the three heptapeptides of interest as it had a minimal effect on the stability of these heptapeptides after 24 (±1) h of incubation.

### Stability of the peptide-chemical complexes when stored in autosampler

Chemical reaction of amino acid residues in the heptapeptides with test chemicals involves irreversible covalent bond formation mimicking the reaction of haptens with amino acid residues of skin proteins (Gerberick et al., 2004). However, a major challenge with the existing peptide reactivity assay method is that the stability of the covalent bond formed between heptapeptides and test chemicals over an extended period, as may be required by high-throughput peptide reactivity assay screening of large batches of chemicals, is unknown. In our present work, we identified the maximum period that sample analysis could be performed accurately based upon the stability of the peptide-test chemical complexes formed. Our data showing that peptide-chemical complex formation appears to be partially reversible in some instances, are novel. For example, following incubation of cinnamaldehyde with Cor1-C420 or the cysteine heptapeptide, apparent peptide depletion decreased by 5 and 13%, respectively, by day 3 following initiation of the peptide-chemical reactions. In these instances, the magnitude of these changes did not alter the skin sensitization classifications. The stability of peptide-test chemical complex formed was assessed against standard QC samples (without test chemical) stored for the same length of time in the autosampler at 4°C. As the concentrations of the heptapeptide standard QC samples remained consistent throughout the course of experiment, this means that any changes observed in apparent levels of peptide depletion during the 3-day storage period in the autosampler to cause a change in the chemical reactivity classification of the test chemicals, were not due to instability of the heptapeptides. Instead, our findings suggest that some of the peptide-chemical complexes were held together by slowly reversible covalent bonds. Indeed, our findings are aligned with similar findings in work by others on the kinetic profiles of test chemical-peptide reactions for periods ranging from 5 min to 24 h post-incubation (Natsch et al., 2011; Roberts and Aptula, 2014; OECD, 2015). Our findings extend previous work to suggest that dissociation of peptide-chemical complexes formation were not ideal for assessing the peptide reactivity assay.

Additionally our data indicate that the peptide-chemical complex dissociation rate is chemical-specific. For example, change in apparent peptide depletion was prominent for the Cor1-C420-cinnamaldehyde complex such that during the first 24 h of complex formation, it was classified correctly as a moderate sensitizer (Gerberick et al., 2005). However, it would have been incorrectly classified as a non-sensitizer if assessed only on day 3 post-incubation. By contrast, the extent of peptide depletion determined following incubation of DNCB with the Cor1-C420 differed by ≤5% over several days of storage at 4°C in an autosampler. For example, cinnamaldehyde was initially assessed as having moderate peptide reactivity when assessed on day 0 which was in line with known LLNA data, with the reactivity gradually decreasing with minimal/no reactivity by 48 h post-chemical incubation (day 1). In addition, following the incubation of isoeugenol and methyl salicylate with Cor1-C420, a marked increase in peptide depletion over the 3-day assessment period, that would lead to misclassification of the sensitizing reactivity of each of these chemicals. Work involving assessment of the kinetic reactivity profiles of test chemicals with the cysteine-containing heptapeptide showed that the extent of cysteine depletion was dependent upon both the test chemical concentration and the incubation time, thereby potentially affecting the chemical potency classification (Roberts and Natsch, 2009; Natsch et al., 2015). Although future investigation is required to characterize the dissociation rate kinetics of peptide-chemical complex formation for a broad range of chemicals, we recommend based upon our present findings showing time-dependent changes in apparent peptide depletion by a range of heptapeptides and chemicals, that all peptide reactivity samples under the experimental conditions chosen here be analyzed within 24 h of initiation of incubation (at 25°C) between the heptapeptides of interest and a test chemical.

Our present research highlights the importance of optimizing the reaction conditions in a systematic and comprehensive manner when evaluating the applicability of an assay such as the peptide reactivity assay for assessing a wide range of chemical classes. It is crucial to determine the choice of peptide for peptide reactivity assay as not all sensitizers will react with thiol and/or amine side chains. For instance, DNCB is thiol reactive and therefore, it binds with the thiol side chain of Cor1-C420 and cysteine-containing heptapeptide. Thus, in skin cells, DNCB will then activate the nuclear factor erythroid-derived 2-related factor 2 (Nrf2)-ARE signaling pathway that has a well-known role in the toxicity pathway activated by skin sensitizers (Natsch, 2010). In contrast, DNCB did not bind with the amine group in lysine, whereas glutaraldehyde is a lysine reactive compound and so was suitable for assessment in our lysine depletion assay. Due to the nature of the chemical reactivity of compounds, peptides with different side chains were included in the peptide reactivity assay as suggested in OCED TG442C.

### MS/MS detection

We used MS/MS herein rather than a UV detector as per the OECD TG442C (OECD, 2015), because MS/MS is more sensitive and selective compared with UV-based detection systems (Natsch and Gfeller, 2008). In addition, the use of MS/MS allows us to measure the adduct formation. However, we did not measure the adduct formation in our high-throughput assay due to different molecular mass for each compound. Use of MS/MS detection enabled us to adapt the peptide reactivity assay to a smaller reaction volume prepared in 96-well plate format. This 96-well assay format improved the assay efficiency where it reduces the total analysis time per sample. It was noted that the total number of substances that can be analyzed in peptide reactivity assay such as DPRA is limited by the maximum analysis time, i.e., 30 h as mentioned in OECD TG442C (OECD, 2015). Therefore, the reduction of analysis time for each sample may increase the total number of substances screened. Although the 96-well plate format provides a crucial step toward high throughput assay, the existing method could not increase the efficiency of the test considering that different peptides are required to test independently. A modified version of peptide reactivity assay where a 96-well plate format with simultaneous readout of all peptides is required to increase the assay efficiency. However, formal validation would be needed with the new variant of the assay.

## Conclusion

In summary, we investigated systematically a number of critical aspects of the peptide reactivity assay that may potentially confound the accuracy and reproducibility of the data generated by the peptide reactivity assay. Use of three different heptapeptides in the peptide reactivity assay has the potential to increase assay specificity for detection of skin sensitizers that may bind more favorably to a particular amino-acid on one peptide rather than another. Hence, optimization of the assay protocol to provide favorable assay conditions for these peptides and the different chemical classes being assessed is recommended to ensure that accurate and meaningful data are obtained from the peptide reactivity assay. More chemical classes will be tested using the suggested parameters in our future study to determine the suitability of this assay. However, it has been noted that it is impossible to optimize a general method so that it is suitable for all chemical classes. For example, our peptide reactivity assay using cysteine- and lysine-containing heptapeptides is not suitable for assessing sensitizing metals as stated in ECVAM (2013). Nevertheless, the inclusion of Cor1-C420 heptapeptides in our peptide reactivity assay could not prevent the formation of the coordination bonds between nitrogen or oxygen atom in the amino acid residues and metal ions. Additionally, our findings show that conduct of the peptide reactivity assay in large batch sizes may result in inaccurate data due to instability of chemical bond formation between heptapeptides and some chemical compounds. These observations further highlight the difficulty in adapting *in vitro* methods to high-throughput formats for screening of large numbers of chemicals whilst ensuring that the data produced are both accurate and reproducible.

## Authors contributions

CW performed the experiments. CW and AL wrote the manuscript. All authors contributed to the design of the experiments. Additionally, all authors reviewed and commented on the manuscript drafts and approved the final draft.

### Conflict of interest statement

The authors declare that the research was conducted in the absence of any commercial or financial relationships that could be construed as a potential conflict of interest.

## Footnotes

^1^Category of test chemical is based on the OECD TG442C (Table 2).

^2^Category of test chemical is based on the OECD TG442C (Table 3).

^3^Category of test chemical is based on the OECD TG442C (Table 2).

## Abbreviations

ACD: Allergic contact dermatitis
ANOVA: Two-way analysis of variance
BSA: Bovine serum albumin
DPRA: Direct peptide reactivity assay
LLNA: Local lymph node assay
LLOQ: Lower limit of quantification
MRM: Multiple reaction monitoring
OECD: Organization Economic Cooperation Development
ULOQ: Upper limit of quantification.
